# Lewy Body Dementias: A Coin with Two Sides?

**DOI:** 10.3390/bs11070094

**Published:** 2021-06-22

**Authors:** Ángela Milán-Tomás, Marta Fernández-Matarrubia, María Cruz Rodríguez-Oroz

**Affiliations:** 1 Department of Neurology, Clínica Universidad de Navarra, 28027 Madrid, Spain; AMILAN@UNAV.ES; 2 Department of Neurology, Clínica Universidad de Navarra, 31008 Pamplona, Spain; mfernandezma@unav.es; 3 IdiSNA, Navarra Institute for Health Research, 31008 Pamplona, Spain; 4 CIMA, Center of Applied Medical Research, Universidad de Navarra, Neurosciences Program, 31008 Pamplona, Spain

**Keywords:** Lewy body dementias, dementia with Lewy bodies, Parkinson disease dementia, diagnosis, biomarkers

## Abstract

Lewy body dementias (LBDs) consist of dementia with Lewy bodies (DLB) and Parkinson’s disease dementia (PDD), which are clinically similar syndromes that share neuropathological findings with widespread cortical Lewy body deposition, often with a variable degree of concomitant Alzheimer pathology. The objective of this article is to provide an overview of the neuropathological and clinical features, current diagnostic criteria, biomarkers, and management of LBD. Literature research was performed using the PubMed database, and the most pertinent articles were read and are discussed in this paper. The diagnostic criteria for DLB have recently been updated, with the addition of indicative and supportive biomarker information. The time interval of dementia onset relative to parkinsonism remains the major distinction between DLB and PDD, underpinning controversy about whether they are the same illness in a different spectrum of the disease or two separate neurodegenerative disorders. The treatment for LBD is only symptomatic, but the expected progression and prognosis differ between the two entities. Diagnosis in prodromal stages should be of the utmost importance, because implementing early treatment might change the course of the illness if disease-modifying therapies are developed in the future. Thus, the identification of novel biomarkers constitutes an area of active research, with a special focus on α-synuclein markers.

## 1. Introduction

Lewy body dementia is an umbrella term that includes Parkinson’s disease dementia (PDD) and dementia with Lewy bodies (DLB), which are two entities on a spectrum of Lewy body disease. The two disorders share many clinical and pathological features, including the deposition of widespread limbic and cortical Lewy bodies and Lewy neurites composed of aggregates of α-synuclein (α-syn) [1] and loss of midbrain dopamine cells and cholinergic neurons in the ventral forebrain nuclei [2].

DLB is the second most common form of dementia after Alzheimer’s disease (AD). However, previous studies have shown a wide prevalence variation, probably due to DLB being underdiagnosed [3]. In a systematic review of twenty-two studies addressing the prevalence and incidence of DLB [4], DLB accounted for 3.2–7.1% of all dementia cases in the incidence studies, and the point and period prevalence estimates increased with age and ranged from 0.02 to 63.5 per 1000 persons. Nevertheless, these numbers probably underestimate the true prevalence and incidence of DLB because misdiagnosis as AD is common [3].

In addition to the common motor manifestations of Parkinson’s disease (PD) (tremor, rigidity, akinesia), other non-motor manifestations, such as sensory abnormalities, autonomic dysfunction, and behavioral and cognitive changes, are common [5,6]. About 25% of patients newly diagnosed with PD fulfill the diagnosis of mild cognitive impairment (PD-MCI) [7,8]; at follow-up (mean follow-up 23.5 ± 10.3 months), 33.3% of the PD cognitively normal patients develop MCI and 4.8% convert to PDD, resulting in an incidence rate of 123.5/1000 per year (95% CI 70.3–202.2) [9]. Most individuals with PD have MCI or dementia with the progression of the disease [10,11,12], which occur in up to 83% of cases after 20 years of disease duration [13].

The diagnosis of PDD and DLB is challenging because many clinical manifestations and additional findings in the examinations overlap. However, reaching an early and accurate diagnosis is critical to disentangle the heterogeneity of these two entities, and to develop proper and specific clinical trials of neuroprotective therapies, thus providing an early optimal treatment and a correct prognosis to patients and caregivers. The purpose of this article is to provide an overview of the main neuropathological findings, clinical features, current diagnostic criteria, principal biomarkers, and management of DLB and PDD.

## 2. Methods

A literature search was performed using the PubMed database for January 1995 to January 2021 using the following disease-specific keywords—“Parkinson disease dementia,” “Lewy body dementia,” “Lewy body disorders” and “Lewy body disease”—together with one of the modality-specific keywords: “neuropathology,” “diagnostic criteria,” “prevalence,” “behavioral features,” “neuropsychiatric symptoms,” “magnetic resonance imaging,” “positron emission tomography,” “single-photon emission computed tomography,” “diffusion tensor,” “diffusion-weighted,” “quantitative susceptibility mapping”, “proton spectroscopy,” “polysomnography,” “electroencephalogram,” and “management.” Acronyms, e.g., “PET” for “positron emission tomography,” were entered as appropriate. Abstracts of studies written in English from 1995 to 2021 were reviewed and those with a small sample number (case reports and case series) were excluded. We included longitudinal cohorts, case-control studies, major reviews, and three ongoing clinical trials.

## 3. Current Diagnostic Criteria for Parkinson’s Disease Dementia and Lewy Body Dementia

Table 1 shows the current diagnostic criteria for PDD and DLB. For the clinical diagnosis of both entities, dementia syndrome must always be present.

Revised clinical diagnostic criteria for DLB were published in 2017 [14]. Major differences with the previous criteria [16] included distinguishing among clinical features and biomarkers; upgrading REM sleep behavior disorder (RBD) from a suggestive feature to a core clinical feature based on interim evidence; downgrading of antipsychotic (neuroleptic) hypersensitivity to a supportive feature based on reducing the frequency in prescribing D2 blocking antipsychotics in clinical practice [17]; and elimination of suggestive feature category. Advances in revised diagnostic criteria for DLB are supposed to improve diagnosis, but the impact of these new criteria is not yet known.

Consensus criteria for PDD were developed in 2007 [15]. PDD is diagnosed in the context of an established diagnosis of PD, and consists of identifying a profile of cognitive and behavioral changes consistent with PDD and excluding other potential factors (Table 1). The criteria require impairment in more than one cognitive domain, and emphasize that non-cognitive features such as hallucinations are common. The presence of at least one behavioral symptom contributes to support the diagnosis of probable PDD; however, the lack of behavioral symptoms does not exclude the diagnosis [17]. Whereas supportive and indicative biomarkers are included in the 2017 revised diagnostic criteria for DLB, current clinical criteria of PDD do not include biomarker information.

Although the progression of the symptoms primarily differentiates PDD from DLB, there are no well-founded reasons to define the time interval between the development of parkinsonism and the onset of dementia. However, to avoid diagnostic confusion in the clinical setting, the Movement Disorders Society Task Force recommends that the diagnosis of PDD should be made when dementia develops within the context of well-established PD, whereas a diagnosis of DLB is more appropriate when the diagnosis of dementia precedes or coincides within one year of the beginning of motor symptoms [15].

More recently, prodromal DLB diagnosis has become more common, and the updated research criteria for prodromal DLB suggests that if the order of parkinsonism and cognitive decline cannot be clearly established, then an initial diagnosis of prodromal DLB may be preferable (see Table 2) [18].

Although there is no DLB-specific assessment battery, some authors have explored suitable instruments, such as a composite risk score tool, to evaluate different clinical manifestations, which showed good outcomes in terms of the receiving operating characteristic curve for DLB vs. AD = 0.93 and for prodromal, MCI-DLB vs. MCI-AD = 0.96 [19]. Neuropsychological assessment serves an important role in providing objective evidence of cognitive impairment to support the clinical diagnosis of dementia in PD and DLB [14,15].

## 4. Diagnostic Approach

**Figure 1:**
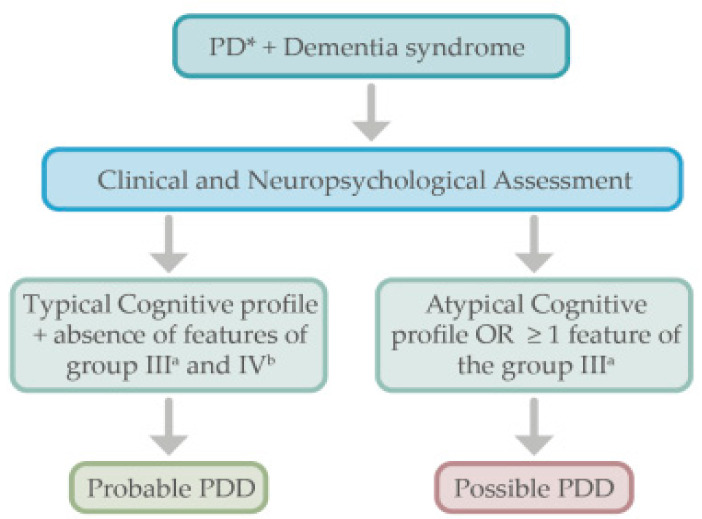
Proposed algorithm for the diagnostic approach in Parkinson’s disease dementia. * Parkinson’s disease diagnosis must be present. ^a^ See Table 1 for the group III features. ^b^ See Table 1 for the group IV features.

PD subjects who develop dementia do not need further complementary examinations as per the PDD diagnostic criteria [15] (see Figure 1). Like in DLB, a comprehensive neuropsychological evaluation including the assessment of specific cognitive domains (attention, memory, executive functions, language, construction, praxis, and visuospatial functions) is useful to assess the specific profile of cognitive impairment.

The diagnosis of DLB is based on the clinical history, physical evaluation, and neuropsychological assessment. When two core clinical features are present, the diagnosis of probable DLB is made, but in the case of only one core feature, the positivity of one or more indicative biomarkers (e.g., DAT-scan or ^123^IMBG) helps in the diagnosis of probable DLB.

**Figure 2:**
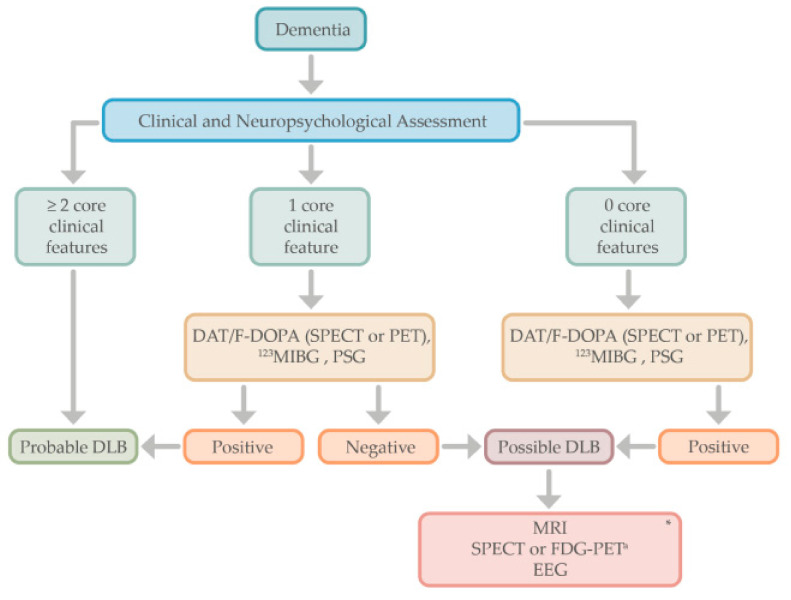
Proposed algorithm for the diagnostic approach in Lewy body dementia. * Supportive biomarkers that help in the diagnostic evaluation but without clear diagnostic specificity. ^a^ The cingulate island sign is only identifiable in FDG-PET, not in SPECT. DLB, dementia with Lewy bodies; DAT; dopamine transporter; F-DOPA, Flurodopa PET; SPECT, single photon emission computed tomography; PET, positron emission tomography; MIBG, iodine-123 –metaiodobenzylguanidine; PSG, polysomnography; MRI, magnetic resonance imaging; FDG-PET, fluoro-deoxy-glucose PET; EEG, electroencephalogram.

Moreover, for those patients in which dementia syndrome does not meet any of the core features of DLB, indicative biomarkers could be performed to establish the diagnosis of “possible” DLB or other neurodegenerative processes such as AD [20,21]. For those patients with atypical findings (e.g., a cognitive profile affecting memory rather than executive or visuospatial dysfunction), or for those patients with dementia in which only one of the core features of DLB is present, the use of functional imaging (e.g., DAT scans) or other indicative biomarkers may help in the diagnosis of probable DLB (see Figure 2). Supportive biomarkers such as FDG-PET, showing the typical “island sign,” or a brain MRI with relative preservation of medial temporal lobe structures, increase the certainty of a DLB diagnosis [14].

## 5. Cognitive Profile of Lewy Body Dementias

MCI is common in non-demented PD patients and the reported relative risk for developing dementia in PD compared to non-PD subjects ranges from 1.7- to 5.9-fold [15]. MCI and dementia in PD is heterogeneous, and combined different profiles (e.g., amnestic and non-amnestic, single versus multiple domains) have been described in PD-MCI [7]. However, from the perspective of progression to dementia, among others, two distinctive phenotypes are recognized: a fronto-striatal/executive pattern, which might be related to dysfunction in dopaminergic fronto-striatal networks [22], and a posterior cortical/visuospatial phenotype, which may involve changes in cholinergic transmission [2,23], which has been shown to be more correlated with cognition decline in LBD [23,24,25].

The impaired cognitive domains in PDD and DLB mostly include attention, memory, visuo-spatial, constructional, and executive functions, predominating the last one in LBD compared to AD [26,27]. LBD patients tend to perform worse in all perceptual scores compared to AD. This is consistent with previous neuroimaging studies reporting hypoperfusion or hypometabolism in cortical areas involving visual processing in PDD and DLB [23,24,25,28,29], which has been included as a supportive biomarker of DLB [14].

Smirnov et al. (2020) [30] published a study focusing on domain-specific patterns of cognitive impairment and trajectories of decline that differ in patients with PDD and autopsy-confirmed DLB. The authors found that DLB and PDD were more impaired and declined more rapidly than in AD in the visuospatial domain, whereas memory was more impaired in AD compared to DLB and PDD. However, this study also observed a greater impairment and more rapid decline of executive function in PDD than in DLB, whereas language and verbal memory dysfunction were more prominent in DLB and AD than in PDD in the late stages of the disease. Although there is also evidence of verbal and non-verbal memory deficits in PDD and DLB [27,31], these are mostly related to executive dysfunction (e.g., observed improvement in recall with cueing, which relates to a retrieval problem rather than to encoding and storage deficits as occurs in AD). However, some authors have shown possible different patterns of verbal learning and memory deficits in PDD versus DLB [32]. This might be due to a higher frequency of concomitant AD pathology in DLB compared to PDD, which may influence the cognitive profile to more closely resemble that of AD in DLB cases.

Cognitive fluctuations with variations in attention and alertness constitute a core clinical feature of DLB. They consist of episodes of spontaneous alterations in cognition, attention, and arousal, leading to frequent daytime drowsiness, naps during the day, or perturbed flow of ideas. Although more frequent and severe in DLB patients [33,34], fluctuations in cognition can also be present in PDD. In fact, “impaired attention which may fluctuate” is included as an associated clinical feature in the diagnostic criteria for the diagnosis of probable and possible PDD. Cognitive fluctuations can be hard to differentiate from toxic-metabolic processes and to quantify [35,36]. Some scales and questionnaires have been proposed for this purpose [36,37]. The updated criteria for the diagnosis of DLB recommend that at least one measure of fluctuations in cognition should be documented when applying its criteria [14].

Regarding the setting of diagnosis, it is worth mentioning that although many cognitive features appear to be similar in DLB and PDD, visuoperceptual and visuoconstructional functions and verbal and visual memory may be worse in DLB compared to PDD [38].

## 6. Behavioral and Neuropsychiatric Manifestations of Lewy Body Dementias

Neuropsychiatric manifestations are common in LBD patients. Depression, anxiety, and apathy are frequent in PDD and DLB [39,40,41,42,43], and they are included as supportive and associated features of both entities (Table 1). They may share a similar substrate, with depression being one of the most studied non-cognitive psychiatric disorders in PD, occurring in up to 50% of PD patients at some point in the course of their illness [42]. Depression is also linked to cognitive impairment [44,45]. Moreover, premorbid depression has been shown to be significantly more common in PD patients than in those without a diagnosis of PD [6,46,47], preceding the motor symptoms and possibly dementia diagnosis of LBD [6,43]. Depression in DLB is also very frequent, with a prevalence of approximately 28% [48], and overall, depression in LBD is more frequent when compared to AD [49].

Apathy, defined by reduced initiative and motivation, appears to be equally common in PDD and DLB, reported in 54% of PDD patients [50] and in 57% of DLB [51]. Moreover, apathy appears to be associated with more serious symptomatology in DLB and faster cognitive decline [52]. Regarding anxiety, comparisons of its prevalence in PDD and DLB are lacking [38], but appear to be worse in DLB compared to in AD [53].

The psychosis spectrum in PD was recently reviewed [54] and includes minor experiences or hallucinations, such as passage and presence hallucinations or illusions in the early stages of PD [40], and well-formed complex recurring visual hallucinations with other modalities of hallucinations and delusions in later PD stages. The presence of cognitive impairment, depression, and dopaminergic use have been described as risk factors for hallucinations [55], and its phenomenology in PDD and DLB is very similar, with the majority of patients experiencing recurrent, complex visual hallucinations—seeing adults or small children, deceased family members, or small animals [1]. Visual hallucinations are usually more frequent and severe in DLB than in PDD (76% vs. 54%) [43], whereas auditory and other modalities of hallucinations are less prevalent in both entities [56,57]. Although hallucinations in PD may be triggered by the use of dopaminergic therapies, they can occur in drug-naïve PD patients [58]; however, they appear more often in DLB spontaneously [39].

A study analyzed the utility of pareidolias, measured by the Pareidolia test, as a surrogate indicator of visual hallucinations in patients with DLB [59]. The authors found that pareidolic responses were observed more frequently in patients with DLB than AD or healthy controls, and were detected even in patients without visual hallucinations. Thus, they proposed that pareidolic responses could be a possible predictive marker of DLB. In other study including PD patients without dementia [60], authors found that the number of pareidolic illusions correlated with hypometabolism in the bilateral temporal, parietal, and occipital cortices, which may suggest that posterior cortical dysfunction could be a common neural substrate of pareidolia and visual hallucinations. However, the test has not been properly assessed in PDD, so its utility as a diagnostic tool is not known.

In summary, although behavioral and neuropsychiatric symptoms are common in both PDD and DLB, most of the studies have shown that some of them, such as visual hallucinations and delusions, are more prevalent and severe in DLB compared to PDD [39,43]. Mood disturbances such as depression or apathy appear to be equally common in both disorders, but these are higher than in AD population. The use of questionnaires and other tools assessing neuropsychiatric symptoms in patients with LBD is relevant to obtain an adequate characterization of these manifestations and may help in the diagnosis.

## 7. From Neuropathology to the Clinical Spectrum of Lewy Body Dementias

PDD and DLB are clinical syndromes characterized by the neuropathological accumulation of misfolded α-syn aggregates that form intraneuronal Lewy bodies and Lewy neurites. Different studies [16,61,62] have reported that cortical or diffuse, or limbic, Lewy bodies (LB) and Lewy neurites correlate well with dementia in PD, indicating a caudal to rostral spread of this pathology from the brainstem to cerebral cortex. Staging systems of α-syn pathology based on these observations have been proposed for PD [63] and DLB [64].

Accumulating evidence indicates that other pathologies are also present in patients with LBD. Superimposed AD-associated neuropathological changes (fibrillary amiloid β-protein, Aβ, and intraneuronal tangles consisting of hyperphosphorilated tau, p-tau) are common in DLB and PDD, with up to 50% of PDD patients showing severe AD-type pathology [65]. Larger study cohorts [65,66,67] have shown a correlation and synergistic effects between both cortical Lewy and AD-type pathologies, with higher cortical Aβ accumulation implying faster progression to dementia [66,68]. A potential mechanism for this synergy is through phosphorylation. α-syn can induce tau hyperphosphorylation, thereby promoting neurofibrillary tangle formation, and vice versa [68,69,70,71,72,73]. Moreover, the frequency and severity of Aβ and tau pathology in the midbrain across the LBD-spectrum have been shown to be located between those of controls and AD, with Aβ in the tectum/tegmentum [74] and the striatum [75] being associated with dementia. Hepp et al. (2016) [67] showed that Aβ pathology was more often observed in the entorhinal cortex, amygdala, and putamen in DLB versus PDD patients. In contrast, PDD patients had Aβ pathology more frequently in the temporal cortex and striatum versus PD patients without dementia, suggesting that the load and extent of Aβ pathology may contribute to cognitive dysfunction in PDD. One of the limitations of these studies is the lack of detailed neuropsychological assessments; therefore, conclusions regarding the relationship between specific cognitive disturbances and the regional prevalence and severity of Aβ pathology are not available.

In patients with PDD, the hippocampus shows a higher density of Lewy pathology [1,76]. Although some authors have suggested that high neocortical and limbic LB burden is the only independent predictor of dementia in DLB [72,73], others considered AD-related pathology to be more important in the decline of cognition in LBD [68,71,72,77,78,79].

A systematic review analyzing the contribution of Aβ deposition (measured by amyloid PET) on the cognition of PD [80] supported the notion of Aβ as an independent predictor of impaired cognition in the setting of PD. Correlations have also been shown between Aβ deposition in multiple cortical regions (e.g., frontal, posterior cingulate, temporal, parietal, and occipital) with lower performance in a test involving attention, working memory, and visual processes, and attentional processing in PD without dementia [81]. From both clinical and pathological perspectives, LBDs are heterogeneous disorders, and different studies indicate that the cognitive decline and related symptoms are not only a consequence of α-syn induced neurodegeneration, but also of mixed pathologies contributing to the overall deficits [68,69,71,82,83].

Despite many similarities between PDD and DLB, some pathological differences have been demonstrated [39,84], including less severe and less extended Aβ load and lower tau load in the cortex and striatum in PDD compared to DLB [39,85,86]; and higher Aβ load in the cortex and claustrum [69,77], and in the entorhinal cortex, amygdala, and putamen in DLB [67]. Moreover, an important contribution of the presence of striatal Aβ in the cognition of PD was shown when compared to cortical β-amyloidopathy alone [75,87]. Other differences include a more severe α-syn load in the amygdala in DLB compared to PDD [88]. In PD, the highest load of α-syn is found in the cingulate cortex, basal forebrain, and hippocampus [69,76,89], with a higher deposition in the claustrum rising progressively from PD without dementia to PDD and DLB [88]. Moreover, hyperphosphorylated tau (p-tau) in LBD has been shown to be significantly lower than in AD but significantly higher than in controls [74], and may display a different pattern in DLB vs. PDD [69]. Global tau indices independently predicted dementia in PD cases in one study [90], whereas in two other studies authors did not find such an association [65,77]. Finally, other co-pathologies, including cerebrovascular disease and TDP-43, are also likely to influence clinical features and progression in LBD [91,92].

## 8. Biomarkers for Lewy Body Dementias

Direct biomarker evidence of Lewy body-related pathology is not yet available for clinical diagnosis; however, several methods have been used to support the clinical diagnosis of PDD and DLB. Neuroimaging techniques, fluid biomarkers, and potential (research) biomarkers are discussed in the following paragraphs. A summary of the main results of these biomarkers in LBD can be seen in Table 3.

### 8.1. Magnetic Resonance Imaging in Lewy Body Dementias

#### 8.1.1. Structural Magnetic Resonance Imaging

Structural brain changes can be assessed using magnetic resonance imaging (MRI) and computed tomography (CT), providing a measure of cerebral atrophy in PDD and DLB. The following MRI sequences are commonly used: T1, T2, T2*, R2* (R2* = 1/T2*)-weighted, susceptibility-weighted, proton-density-weighted, fluid-attenuated inversion recovery, and neuromelanin-sensitive sequences.

Voxel-based morphometry (VBM) studies in PDD have identified diffuse and heterogeneous patterns of cortical atrophy involving the occipital, temporal, right frontal, and left parietal lobe in comparison to controls and with PD without dementia [93,94,95,96], but they could be part of a common brain network centered on the hippocampi as per lesion network mapping [97]. Cortical thickness in the right precentral, superior frontal gyri, and the anterior cingulate cortex [98], and less gray matter (GM) volume in the prefrontal areas, insular cortex, and caudate nucleus [99,100], together with hippocampal atrophy, were observed in PD patients who developed dementia during follow-up [101]. VBM and cortical thickness has also been evaluated in DLB compared to AD, healthy controls, and PDD [102,103,104,105,106,107,108]. Although localization of GM reductions in DLB relative to PDD vary among different studies, Beyer et al. (2007) [93] reported greater GM reductions in the temporal, parietal, and occipital lobes in DLB compared to PDD. In addition to temporal and parietal atrophy, occipital and striatal GM reductions in DLB have also been reported [102].

Studies focusing on prodromal cases of LBD have shown that preserved hippocampal volumes are associated with an increased risk of probable DLB competing with AD dementia, which could help in the differential diagnosis of both entities [108], and it has been included as a supportive diagnostic feature of DLB [14]. In the case of PD, low hippocampal volume was described as a major factor predicting the development of mild cognitive impairment and dementia [101].

Diffusion tensor imaging (DTI) has shown widespread reduced fractional anisotropy (FA) in PDD when compared with cognitively normal PD patients and controls, compromising the main tracts (the superior and inferior longitudinal, inferior fronto-occipital and uncinate fasciculi, the cingulum, the corpus callosum, corona radiata, the anterior limb of the internal capsule, and the hippocampus) [109,110,111,112,113,114]. Cognitive performance strongly co-related to DTI metrics in the most anterior (projecting to the prefrontal cortex) and most posterior (callosal) sections, which may contribute to “fronto-striatal” and “posterior cortical” types of cognitive deficits seen in PD, respectively [115]. In DLB, increased mean diffusivity (MD) was found to be similar to AD and included clusters in the bilateral parahippocampal gyri (hippocampal cingulum) and left cingulate gyrus (frontal), but no correlation between FA and episodic memory in AD or DLB was found [116]. Other studies have evaluated the changes of FA in PD and DLB with and without hallucinations [117,118], and comparing DLB to AD [119]. However, the majority did not compare PDD with DLB [120], with one study showing that FA in patients with DLB was significantly decreased in bilateral posterior temporal, posterior cingular, and bilateral visual association fibers extending into occipital areas (*p* < 0.001) [121].

Changes in the substantia nigra pars compacta (SNpc) occur early in the disease process of parkinsonism-related disorders. The increase in iron content, or loss of paramagnetic neuromelanin–iron complexes containing neurons in the nigrosome-1 (the caudal portion of the SNpc), has been explored as a possible biomarker and target in parkinsonian disorders [122]. These alterations can be seen on susceptibility-weighted imaging (SWI), also known as the loss of the “swallow tail sign.” This sign has been explored in prodromal stages of α-synucleopathies such as RBD [123], and has shown good sensitivity and specificity in the differentiation of PD from controls [124]; in the diagnosis of DLB, sensitivity, specificity, and accuracy were 80%, 64%, and 73%, respectively, when compared to AD [125]. This sign was not able to distinguish between PD and other Parkinson-Plus syndromes [126], and studies comparing PDD and DLB patients are lacking.

MRI quantitative susceptibility mapping (QSM) is a novel technique that can quantify the magnetic susceptibility value of brain tissue from gradient-echo (GRE) MRI data, and can more precisely measure the iron deposition compared to those using SWI and transverse relaxation rate (R2*) mapping [127]. Some studies have reported abnormal iron deposits in some deep brain nuclei of PD patients, including the red nucleus, caudate nucleus, globus pallidus, putamen, thalamus, and dentate nucleus [127,128,129,130], and in the SN [131,132], but the results are not completely consistent. Studies evaluating the correlation between cognitive impairment in PD and the load of iron content [132,133] have identified that QSM increases covary with (1) MoCA scores in the hippocampus and thalamus; (2) poorer visual function and higher dementia risk scores in parietal, frontal, and medial occipital cortices; and (3) higher UPDRS-III scores in the putamen (all *p* < 0.05) [133]. QSM has also been used in AD [134], showing positive associations between susceptibility and amyloid PET in the pallidum and putamen, but with variable results regarding cortical areas. Overall, these findings suggest that QSM may be useful to track cognitive changes in PD; however, no studies analyzing patients with DLB are available [135].

#### 8.1.2. Functional Magnetic Resonance Imaging

Resting-state and task-based functional MRI using perfusion or, more typically, by measuring blood-oxygenation-level-dependent (BOLD) signals using T2*-weighted have identified multiple networks involving motor and non-motor circuits in PD and DLB.

As in the case of other techniques, the majority of studies evaluate changes of fMRI comparing PDD and DLB against controls or AD [120], but comparisons between PDD and DLB are scarce.

The abnormal integrity of the dorsal attention network and the involvement of its aberrant nodes in working-memory tasks and visual attention in PD has been reported [136,137]. Most groups demonstrated relatively preserved default mode network connectivity [138,139,140], and disturbed frontoparietal networks and disconnection of the occipital brain regions in DLB compared to healthy controls [139,141]. Although few studies found correlations between FC results and motor/cognitive outcomes [139,142,143], most studies did not observe any significant correlations with clinical outcomes after correction for multiple testing. Overall, functional connectivity (FC) in DLB compared to healthy controls, PDD, and, in particular, to AD groups, remains inconclusive to date. The few existing studies of fMRI with a small sample size did not find significant differences between PDD and DLB [95,140,143].

### 8.2. Nuclear Medicine and Molecular Imaging in Lewy Body Dementias

Single-photon emission computed tomography (SPECT) and positron emission tomography (PET) are well-recognized tools to assess function, and even to evaluate in vivo brain pathology (e.g., amyloid PET). Novel radiotracers are emerging for the study of other specific protein species, and the role of these nuclear medicine tools ranges from early research to clinical diagnostic applications used in the clinical setting.

**Figure 3:**
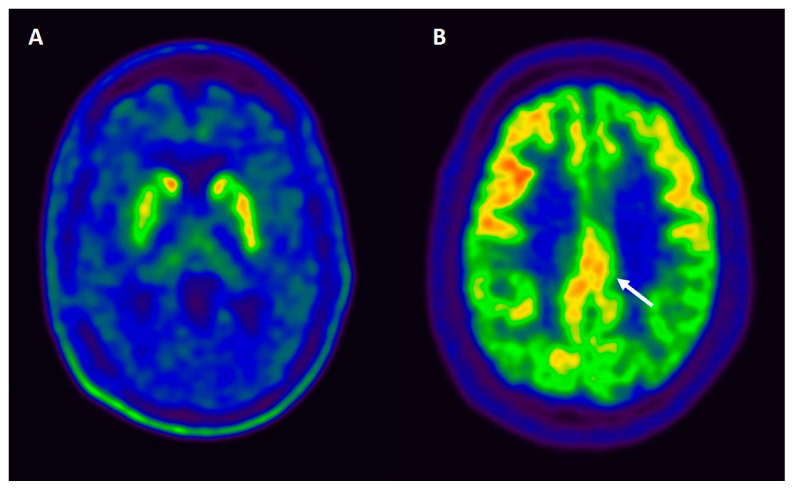
(**A**) Axial ^18^F-Dopa PET image of a patient with dementia with Lewy bodies. The image shows a moderate decrease of bilateral striatal activity (dopaminergic denervation), more pronounced in the right putamen. (**B**) FDG-PET findings in a patient with dementia with Lewy bodies. Axial FDG-PET image demonstrates the cingulate island sign (indicated with an arrow), which reflects preservation of posterior cingulate metabolism relative to cuneus and precuneus.

Loss of the neurons in SN is extensive and characteristic for PD and DLB, and leads to a substantial reduction in the striatal presynaptic dopaminergic function showed by a reduction on presynaptic dopamine transporter (DAT) or 18F-dopa uptake [144] (see Figure 3A). Reduced DAT uptake in basal ganglia demonstrated by SPECT or PET imaging has shown to be useful in distinguishing DLB from AD, based on sensitivity (78%) and specificity (90%) [145]. Moreover, some studies have evaluated the sensitivity of DAT in distinguishing prodromal stages of Lewy body disorders from AD, with a sensitivity of 54% and specificity of 89% [20]. Although an abnormal DAT scan supports the diagnosis of DLB [14], a normal scan does not exclude DLB altogether, including those cases that present with minimal motor symptoms [146], and does not distinguish between PDD and DLB.

Cerebral perfusion SPECT evaluates the metabolic status of brain tissue by quantifying changes in the regional cerebral blood flow. Occipital hypoperfusion is frequently observed in DLB; however, it was unable to differentiate PDD cases from DLB, revealing similar perfusion profiles in some studies [147]. Occipital hypometabolism combined with a less prominent metabolic decrease in the medial temporal lobe using F-18 fluoro-deoxy-glucose (FDG) PET may be useful in differentiating DLB and PDD from AD [148]. Moreover, the relatively preserved metabolism in the posterior cingulate cortex in DLB, also known as the “cingulate island” sign, achieved the highest sensitivity (100%) in differentiating DLB from AD, and is also included as a supportive biomarker in the diagnostic criteria of DLB [14] (see Figure 3B). A study directly comparing the accuracy of FDG-PET with SPECT perfusion found that the cingulate island sign was only present with FDG-PET imaging in DLB, but not with SPECT [149]. Patients with PDD showed large areas (occipitoparietal > frontal) of coincidental hypometabolism and atrophy, and the hypometabolism in PD appears to predate and is replaced by atrophy, in a progressive manner as the cognitive state worsens [150]. PDD and DLB often show similar patterns, but a more prominent hypometabolism in the anterior cingulate cortex may distinguish DLB from PDD [148]. The evaluation of the posterior cingulate cortex in PDD has not received attention compared to DLB, in which the preserved metabolism of this area (the cingulate island) has a high sensitivity and specificity in the differential diagnosis from AD [151,152].

**Figure 4:**
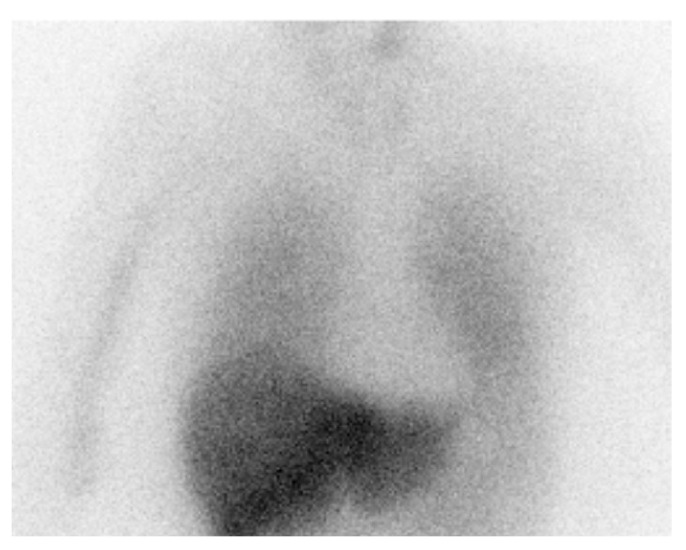
^123^I-MIBG myocardial scintigraphy image taken 3 h after injection. The image shows reduced uptake on ^123^I-MIBG in the heart, indicative of dysfunction in the postganglionic sympathetic cardiac innervation.

Abnormal cardiac uptake of ^123^I-metaiodobenzylguanidine (^123^I-MIBG) is a diagnostic marker of LBD [153]. Prior findings suggest that cardiac sympathetic function in DLB is severely impaired even in the early disease stage, with the uptake of [^123^I]MIBG being significantly lower than that in patients with PD without dementia [154] (see Figure 4). ^123^I-MIBG scintigraphy is useful to distinguish PD and DLB from other diseases [155], and to discriminate between DLB and AD [21,156] and other parkinsonian disorders [157]. However, specific data comparing DLB and PDD are not available and ^123^I-MIBG imaging is unlikely to differentiate PDD from DLB [39,158]. Given its diagnostic accuracy in differentiating DLB from AD, MIBG scintigraphy has been included as an indicative biomarker in the current DLB diagnostic criteria [14]. However, false positive MIBG scintigraphy results due to age, medications, and comorbidities such as diabetes or thyroid dysfunction may limit its use as a routine clinical technique [159].

Finally, PET scans visualizing the deposition of different proteins in the brain (e.g., PET amyloid quantifying Aβ deposition) have been used to evaluate correlations between cognitive impairment in PDD and DLB [160,161].

The apparent gradient of increasing amyloid pathology visualized on PET can be conceptualized as PD < PD-MCI < PDD < DLB [160]. Studies in PDD and DLB have shown higher rates of amyloid-positive scans than in a normal population [160] and are most consistently associated with worse global cognition in LBD [80,160,161,162,163,164], which matches with the findings of pathology studies [67,74,165]. When analyzing prodromal stages of LBD, a recent study found that about one-third of the patients had positive amyloid scans and signs of neuronal dysfunction (measured by hypoperfusion in SPECT scans) early in the disease [166]; this group was older and had worse cognition than the amyloid-negative group. The positivity of Aβ in MCI-LBD was lower than the ratio reported in probable DLB, which could be due to different stages of brain Aβ accumulation over time in patients with probable DLB. This was shown in the longitudinal study of Nedelska et al. (2019) [167], where probable DLB patients with higher baseline and changes in Aβ measured by standardized uptake value ratios (SUVRs) were associated with more rapid clinical and cognitive decline over time.

PET using tracers to bind tau protein remains a research tool. The burden of cerebral neurofibrillary tangles, in addition to α-syn and Aβ pathology, contributes to the motor and cognitive decline in LBD [66,168,169,170]. Tau-PET uptake has been observed in patients with PDD and DLB compared to healthy controls [171], and a greater tau uptake in the inferior and lateral temporal gyri and precuneus of DLB and PD-MCI was found compared to PD without cognitive impairment and controls [162]. Although the correlation found between Aβ and tau uptake in the posterior temporoparietal and occipital cortex suggest that DLB patients are associated with AD-related pathology [172], a significant tau burden was present despite minimal amyloid in a small DLB group, suggesting that extensive tauopathy is possible without amyloid deposition in LBD. However, longitudinal studies with bigger samples are needed to clarify the temporal relationship between increased tau uptake and amyloid deposition in DLB [171,172].

Finally, some potentially labeled radiotracers binding α-syn and neuroinflammation changes in the brain have been studied in recent years, but their use is still under investigation.

There are many challenges in the development of an α-syn tracer, including the low concentrations of the protein compared to Aβ, predominant intracellular location of α-syn, and off-target binding [173]. Several radiolabeled tracers for α-synucleinopathy imaging have been explored [173,174]. However, studies evaluating these tracers failed to show accurate binding of α-syn aggregates in PDD and DLB [175], and more research is needed to evaluate new tracers with higher affinity binding [176].

Neuroinflammation has also been studied in vivo using PET imaging to evaluate microglial activation (as the innate immune response to invading pathogens) in both PDD and DLB [177]. One widely used PET ligand for imaging neuroinflammation is ^11^C-PK11195, which binds to the translocator protein (TSPO), located on the outer mitochondrial membrane in microglia, and has been evaluated in LBD [178,179,180,181,182,183,184]. Compared to controls, increased ^11^C-PK11195 binding in PDD and PD without dementia [182,183] and DLB [177,179] has been observed. Moreover, a significant inverse correlation between levels of microglia activation and glucose metabolism in temporoparietal regions was found in a small sample of PDD and AD, suggesting a deleterious effect of microglia on neuronal function in these dementias [182]. However, there is high variability in the results of clinical studies in PD, which may be due to differences in the evaluated cohorts, the methods of analysis [181], and very small samples [179].

### 8.3. Molecular Biomarkers in Fluids in Lewy Body Dementias

#### 8.3.1. Cerebrospinal Fluid

Different proteins have been investigated as biomarkers for cognitive decline in dementing disorders, namely, cerebrospinal fluid (CSF) Aβ-42 protein (Aβ_42_), total tau (t-tau), and p-tau, which are well established in the diagnosis of AD [185]. Low CSF Aβ_42_ levels have been shown to be related to the development of cognitive impairment in PD and DLB [98,109,186,187,188,189]. Steenoven et al. (2016) [190] compared AD CSF biomarkers (Aβ_42_, t-tau, and p-tau) in PD without dementia, PDD, and DLB. Authors found that a large proportion of DLB patients had abnormal values (AD characteristic pattern), whereas in PD without dementia it was uncommon to find these abnormalities and PDD patients had values between the two. Different profiles of CSF Aβ reduction have been described in DLB and AD, with AD showing an isolated drop in Aβ_42_, whereas DLB exhibited reductions in Aβ_38_, Aβ_40_, and Aβ_42_ [191]. An oxidized α-helical form of Aβ_40_ peptide was found to be significantly increased in patients with DLB in comparison to PDD, with a sensitivity of 81% and specificity of 71% for discriminating among DLB and PDD [192].

CSF levels of total α-syn have been found to be lower in DLB and PD compared to controls and AD [193,194,195,196,197]. Real-Time Quaking-Induced Conversion (RT-QuIC) is an ultrasensitive technique able to detect α-syn seeding activity across the spectrum of LBD with high sensitivity (95.3%) and specificity [198] in distinguishing α-synucleinopathies from non-α-synucleinopathies (including patients with mixed pathology, e.g., AD and DLB) [199].

Longitudinal changes in CSF α-syn were also examined in PD cohorts: two studies showed increasing CSF levels over time [193,200], one reported a decrease [201], and another more recent study showed no longitudinal effects [202]. The study of Mollenhauer et al. (2019) [203] showed that CSF α-syn decreases early in the disease, preceding motor PD, but does not correlate with progression. Therefore, additional biomarkers or their combination (e.g., ratio of p-tau/α-syn and p-tau/Aß1-42+α-syn in PD) have been proposed [204]. Considering the different species of α-syn, CSF levels of oligomeric α-syn were higher in DLB and PD compared with AD and controls [205], and oligomeric and phosphorylated α-syn were also increased in PD compared with controls [206]. Moreover, one study showed that CSF α-syn improved the differential diagnosis between AD and LBD at prodromal stages [207], but overall α-syn in CSF does not appear to help in the differential diagnosis of PDD and DLB [208]. The combination of CSF α-syn with other biomarkers and symptoms might provide more information; however, this remains a matter of research [197].

#### 8.3.2. Other Biological Fluid Biomarkers

α-syn has been analyzed in multiple peripheral tissues, such as saliva and plasma, using diverse techniques such as enzyme-linked immunosorbent assay, Western blot, mass spectrometry, or Luminex© assay [209]. Oligomeric α-syn has been also visualized in the serum and red blood cells of PD patients [210]. RT-QuIC assays have been also performed on other easily obtainable tissues, such as the olfactory mucosa, in patients with DLB [211] and with isolated RBD (as an early-stage α-synucleinopathy) [212], showing a diagnostic accuracy for the clinical diagnoses of DLB of 86.4% for the olfactory mucosa and 93.8% for CSF. These results suggest that nasal swabbing might be considered a first-line screening procedure in patients with suspected DLB, followed by CSF analysis in case of incongruent result with the initial clinical diagnosis [212]. Thus, the development of new techniques allowing the measurement of these biomarkers with less invasive procedures are among the most promising diagnostic approaches.

Another potential biomarker that has been examined in CSF and plasma is the neurofilament light chain protein (NfL) [213]. NfL is a biomarker of axonal damage and its levels have been measured in different neurodegenerative disorders [214]. Plasmatic levels of NfL are higher in PDD than in PD with preserved cognition [215]. In addition, plasmatic levels of NfL showed a good correlation with cognitive function, but not with motor function in 49 PD patients [215]. NfL was found to be elevated in DLB, but no significant differences have been observed in comparison with other dementias. Therefore, NfLs appear to provide only a general hint of neuronal and axonal degeneration, without a differential value for separating DLB from other disorders [213,216].

The role of neuroinflammation in the pathogenesis of neurodegenerative disorders is increasingly being recognized. Inflammation may be involved early in the cognitive decline of patients with MCI due to AD and DLB, and possibly less prominent in PD, as noted per a study measuring different cytokines, such as interferon gamma, interleukin (IL)-10, IL-12p70, IL-13, IL-1beta, IL-2, IL-4, IL-6, IL-8, tumor necrosis factor alpha, and high-sensitivity C-reactive protein [217]. Other studies have found similar results, showing an increase in inflammatory factors in patients with possible and probable AD and DLB in the prodromal stages [177,218,219], although these factors probably do not help in distinguishing subtypes of MCI (due to AD vs. DLB or PD) [218].

Among other fluid biomarkers, vitamin D, lipids, and neurotrophic factors have been explored in LBD and in dementia overall [109,220,221,222,223,224,225], in addition to synaptic and cytosolic proteins, circulating mitochondrial DNA, and fatty acid binding protein 3 [109,186,226,227]. For instance, low uric acid has been shown to be possibly involved in the occurrence of LBD and cognitive decline [228,229]. However, the results of these studies are not robust and need further investigation in larger samples.

### 8.4. Neurophysiological Biomarkers

Evidence is building to support quantitative electroencephalogram (EEG) as a DLB supportive biomarker, characterized by specific abnormalities in posterior derivations with prominent posterior slow-wave EEG activity and periodic fluctuations in the pre-alpha/theta range [14,230,231,232,233]. This specific EEG pattern is included as a supportive biomarker of DLB [14], and also correlates positively with the severity of clinically observed cognitive fluctuations [232], and may be seen at the MCI stage [234].

Sleep disturbance is common in dementia and changes in the architecture of sleep, especially those seen in REM, have been related to the incidence of dementia [235]. The confirmation of REM sleep without atonia using a polysomnography, in conjunction with one or more clinical features, is sufficient for the diagnosis of DLB. RBD is included as a core clinical feature because it occurs frequently in autopsy-confirmed cases compared with non-DLB (76% vs. 4%) [236].

## 9. Evolution and Prognosis of Lewy Body Dementias

It is relevant to note that, although the clinical and pathologic features of PDD and DLB may overlap, the presentation and natural course are usually different. This is important to provide the most accurate information regarding the prognosis and evolution of these entities to the patients and caregivers. In patients with DLB, parkinsonian symptoms appear on average two years after estimated dementia onset [237]; this is in contrast to PDD patients, who must have a well-established diagnosis of PD before dementia begins. Motor manifestations are often more severe in PDD than in DLB; however, DLB patients may respond less to medications [159]. In comparison with DLB, PDD patients commonly have greater motor disability and higher medication burden. Once dementia appears in PD, the prognosis is assumed to be poor, although few studies have focused on the rate of cognitive decline or mortality after dementia onset [238]. Patients with PDD are on average younger than patients with other kinds of dementia, but they have more comorbidity and take more medications, which might increase their mortality compared to other populations [238]. Some of the factors that influence mortality are male gender, a higher number of medications, institutionalization, and age [238]. Age has consistently been demonstrated to be an important predictor of both dementia and mortality in LBD [239].

The course of DLB generally shows a more rapid cognitive decline compared to AD or PDD [240], with an average survival time in DLB being 1.60 years shorter than that in AD [241]. In a retrospective cohort of DLB patients [242], authors found that an amnestic cognitive profile conferred a worse outcome. Furthermore, there is an emerging consensus for the role of concurrent pathologies (e.g., concomitant AD pathology) accelerating cognitive decline in DLB and PDD [66,67,91,241], and other factors such as neuropsychiatric symptoms (especially aggression and psychosis) have been shown to trigger institutionalization, leading to poorer outcomes [241].

## 10. Management of Lewy Body Dementias

Currently, there are no effective therapies that modify the course of LBD. Thus, treatment of both PDD and LBD is mainly focused in managing the more prominent symptoms of the disease (e.g., motor and neuropsychiatric). The management of PDD and DLB is specifically covered in another chapter of this Special Issue, so we will summarize the main therapeutic strategies used in LBD.

One of the first steps is to assess possible harmful medications that could worsen cognitive or motor symptoms in LBD (e.g., benzodiazepines, opiates, anticholinergic medications, or anti-dopaminergic drugs) [159,243].

Evidence supports the use of cholinesterase inhibitors (ChEIs) to treat cognitive and neuropsychiatric symptoms in LBD [244,245]. Moreover, a meta-analysis and trial sequential analysis showed that both ChEIs and memantine improve clinical global impression; however, only ChEIs enhanced cognitive function [244]. Both donepezil and rivastigmine are recommended ChEIs for DLB [246], whereas rivastigmine is the only cholinesterase inhibitor FDA-approved for use in PDD. The evidence for the third ChEI, galantamine, in LBD is scarce because only open-label trials support its use [244]. Clinical trials of the NDMA receptor antagonist memantine showed that it was well tolerated in patients with both PDD and DLB, but evidence for its efficacy remains mixed [244,246,247,248]. Therefore, further studies with larger numbers are needed to determine its use as a monotherapy or combined with ChEIs [245].

Regarding the management of motor symptoms in LBD, dopaminergic replacement therapy is used. Dopamine agonists are avoided as they may worsen cognitive and behavioral symptoms, especially in DLB, so low doses of levodopa as a monotherapy is the most common approach in these cases [159]. Motor function appears to improve more in PDD patients (65–70%) than in those with dementia with Lewy bodies (32–50%) [247]. However, one in three patients with DLB treated with levodopa will experience psychotic symptoms, which need to be taken into account [249]. A meta-analysis of four double-blind randomized controlled trials, including PD [250] and DLB patients [251], reported a significant improvement in motor function with zonisamide, an antiepileptic agent, when used as adjunctive treatment to levodopa.

As per the management of neuropsychiatric symptoms in LBD, non-pharmacological approaches are considered to be first-line strategies (e.g., environmental modifications or musical therapy). However, evidence supporting their efficacy is not robust [252]. Non-bothersome hallucinations and delusions may not require pharmacological treatment. However, disturbing psychotic symptoms could require the use of antipsychotics. Although there is not enough evidence to support the use of any particular antipsychotic drug over others, quetiapine appears to have the fewest side effects in patients with LBD, but evidence for its efficacy in patients with PD [253] and those with Lewy body dementia is insufficient [247,254]. Quetiapine and clozapine, as the remainder of the atypical antipsychotics, carry the risk of weight gain and metabolic syndrome. Their use is of particular concern in individuals with DLB given the risk of hypersensitivity reactions to neuroleptics (e.g., sudden deterioration in motor symptoms and/or mental status changes, such as confusion or even unresponsiveness) [159].

Pimavanserin is a selective serotonin (5-HT) 2A receptor inverse agonist and the US Food and Drug Administration (FDA) approves it only for PD psychosis. A meta-analysis of 13 randomized placebo-controlled trials in PD including Pimavanserin, clozapine, olanzapine, and quetiapine showed that Pimavanserin was associated with a significant improvement in psychotic symptoms compared to a placebo, without worsening motor function. Clozapine was efficacious in alleviating psychotic symptoms and did not exacerbate motor function, but quetiapine and olanzapine did not demonstrate significant differences in reducing psychotic symptoms and may aggravate motor function. Therefore, more well-designed trials confirming these results are needed. There is an ongoing clinical trial evaluating the use of Pimavanserin in dementia-related psychosis, also including DLB (NCT03325556).

Among other non-motor symptoms, sleep disturbances are frequently present in LBD. Melatonin has been shown to be safe and well tolerated, and is considered to be the first-line agent for treating RBD in patients with LBD [159]. Additionally, clonazepam is often tried if melatonin is not sufficiently effective [255].

## 11. Discussion

DLB and PDD are clinically and neuropathologically similar entities distinguished based on the timing of dementia and parkinsonism. Overlapping clinical features and supportive biomarker findings in PDD and DLB complicate their differential diagnosis. Diagnosis is mainly determined based on history, examination, and neuropsychological and neuropsychiatric assessment.

Some characteristics of the cognitive profile and behavioral symptoms, such as greater impairment and more rapid decline of executive function in PDD than DLB, or more severe visuospatial deficits and visual hallucinations in DLB than PDD, may differ between these two entities but they are not useful to discriminate between on an individual basis.

The controversy persists regarding whether DLB and PDD represent two distinct nosological entities or whether they exist on a clinicopathological spectrum of the same disorder. Smirnov et al. (2020) [30] showed that even if DLB and PDD may be nearly indistinguishable pathologically, the two may differ cognitively in the progression of the disease (e.g., visuoperceptual and visuoconstructional functions and verbal and visual memory may be worse in DLB), adding to the current debate on whether the conditions should be pooled or treated separately. A shift to a pathologic classification (e.g., LBD-dementia or LBD-parkinsonism) has been proposed and could become more relevant if α-syn biomarkers are developed.

Different pathological studies have shown that the manner in which α-syn is topographically spread (transitional vs. diffuse Lewy body disease) and other synergistic actions (Aβ and tau co-pathology) may influence the clinical presentation and therefore the diagnosis received early in the disease process. The clinical differentiation between DLB and PDD is still based on an arbitrary distinction between the time of onset of parkinsonism and cognitive symptoms (the one-year rule). However, due to the recent effort made by investigators in developing updated diagnostic criteria for the prodromal stages in DBL and the ongoing research of reliable biomarkers for identifying patients in the early stages of the disease, these diagnostic criteria may change in the near future.

The clinical diagnosis in prodromal stages should be of the utmost importance because, like in other neurodegenerative disorders, early and proper management of the disease might change the evolution and outcomes of the illness. The identification of novel biomarkers is another area of active research. As we have shown in this article, multiple biomarkers could be used in clinical practice to help clinicians in the diagnosis of LBD. Indeed, supportive and indicative biomarkers are included as part of the diagnostic criteria of DLB, whereas the current clinical criteria for PDD do not include biomarker information.

Numerous other novel biomarkers are in development which might change the landscape of in vivo LBD diagnosis. It is likely that the combination of a variety of techniques will provide more accurate results than when each is used separately. We have proposed an algorithm for the diagnosis workup in PDD and DLB (see Figure 1 and Figure 2), which could be used in clinical settings. Among other approaches, the use of FDG-PET, DAT-scan or ^18^F-Dopa PET, and scintigraphy [^123^I]MIBG, can be useful to distinguish LBD from other neurodegenerative processes such as AD. The evaluation of CSF biomarkers might add additional value to the diagnosis process, particularly if new techniques allow us to accurately measure α-syn. The focus on α-syn pathology has both diagnostic and therapeutic implications in the development of disease-modifying treatments. There is a need for a suitable PET radiotracer for imaging of LB pathology, which would represent a valuable tool for that purpose.

The current treatment of LBD remains symptomatic and consists of avoiding medications that may cause or exacerbate symptoms, in addition to other pharmacological and non-pharmacological therapies to alleviate the symptoms of PDD and DLB patients. Clinical trials are ongoing with new molecules that may modify the course of LBD [256] (e.g., ambroxol, NCT02914366, and bosutinib, NCT03888222).

Patient selection approaches for investigational studies focused on cognitive deficits in DLB and PDD may consist of excluding patients without physiological evidence of these diseases, or enriching the mechanism of action or phenotype of interest [257]. In 2019, the Lewy Body Dementia Association (LBDA) launched the Industry Advisory Council to provide a collaborative forum for discussion among LBD experts, pharmaceutical industries, governmental agencies, and the non-profit LBDA to address challenges and opportunities for LBD clinical trials [256]. Refining diagnostic accuracy and continued exploration of the use of clinical and potential research biomarkers is needed to reduce heterogeneity in clinical trials and improve the landscape of DLB.

## Figures and Tables

**Table 1:** Criteria for the clinical diagnosis of probable and possible dementia with Lewy bodies (DLB) and Parkinson’s disease dementia (PDD).

DLB ^1^		PDD ^2^	
Central features	Essential for a diagnosis: Dementia, in early stages with memory impairment, may not necessarily occur but is usually evident with progression. Deficits in tests of attention, executive function, and visuoperceptual ability may be especially prominent and occur early.	Core Features (I)	Essential for a diagnosis (both must be present): Diagnosis of Parkinson disease according to Queen Square Brain Bank criteria and Dementia syndrome with impairment in more than one cognitive domain
Core clinical features	The first three typically occur early and may persist throughout the course: Fluctuating cognition with pronounced variations in attention and alertness. Recurrent visual hallucinations that are typically well formed and detailed. REM sleep behavior disorder (may precede cognitive decline).	Associated clinical features (II)	Typical profile of cognitive deficits (at least two of the four core cognitive domains): impaired attention (which may fluctuate), executive, and visuo-spatial functions, and impaired free recall memory, which usually improves with cueing. The presence of at least one behavioral symptom (apathy, depressed or anxious mood, hallucinations, delusions, or excessive daytime sleepiness) supports the diagnosis of probable
	4. One or more spontaneous cardinal features of parkinsonism: bradykinesia, rest tremor, or rigidity.		PDD; lack of behavioral symptoms, however, does not exclude the diagnosis
Supportive clinical features	Severe sensitivity to antipsychotic agents; postural instability; repeated falls; syncope or other transient episodes of unresponsiveness; severe autonomic dysfunction, e.g., constipation, orthostatic hypotension, urinary incontinence; hypersomnia; hyposmia; hallucinations in other modalities; systematized delusions; apathy, anxiety, and depression.	None of the group (III) features present	Features which do not exclude PDD, but make the diagnosis uncertain: Co-existence of any other abnormality which may by itself cause cognitive impairment, but judged not to be the cause of dementia, e.g., presence of relevant vascular disease in imaging Time interval between the development of motor and cognitive symptoms not known
Indicative biomarkers	Reduced dopamine transporter uptake in basal ganglia demonstrated by SPECT or PET. Abnormal (low uptake) 123iodine-MIBG myocardial scintigraphy. Polysomnographic confirmation of REM sleep without atonia.	None of the group (IV) features present	Features suggesting other conditions or diseases as cause of mental impairment, which, when present, make it impossible to reliably diagnose PDD: 1. Cognitive and behavioral symptoms appearing solely in the context of other conditions such as acute confusion due to (a.) systemic diseases or abnormalities (b.) drug intoxication 2. Major Depression according to DSM IV
Supportive biomarkers	Relative preservation of medial temporal lobe structures on CT/MRI scan. Generalized low uptake on SPECT/PET perfusion/metabolism scan with reduced occipital activity +/− the cingulate island sign on FDG-PET imaging. Prominent posterior slow-wave activity on EEG with periodic fluctuations in the pre-alpha/theta range.	Supportive or indicative biomarkers	No supportive or indicative biomarkers are needed for the diagnosis of PDD as per Emre et al. (2007) diagnostic criteria.
Diagnosis of probable or possible DLB	Probable: (a) ≥2 core clinical features of DLB are present, with or without the presence of indicative biomarkers, OR (b). Only one core clinical feature is present, but with ≥1 indicative biomarkers. Probable DLB should not be diagnosed on the basis of biomarkers alone. Possible: (a). Only one core clinical feature of DLB is present, with no indicative biomarker evidence, OR (b). ≥1 indicative biomarkers is present but there are no core clinical features.	Diagnosis of probable or possible PDD	Probable: (a) Core features: Both must be present; (b). Associated clinical features: Typical profile of cognitive deficits and the presence of at least one behavioral symptom (lack of behavioral symptoms, however, does not exclude the diagnosis); (c) None of the group III features present; (d) None of the group IV features present. Possible: (a) Core features: Both must be present (b). Associated clinical features: Atypical profile of cognitive impairment in one or more domains (e.g., fluent aphasia, or pure storage-failure type amnesia) and behavioral symptoms may or may not be present; OR (c) One or more of the group III features present, (d) None of the group IV features present

(1). Adapted from McKeith, et al. [14]. (2). Adapted from Emre, et al. [15]. DLB, dementia with Lewy bodies; PDD, Parkinson’s disease dementia; MIBG, iodine-123–metaiodobenzylguanidine; CT, computed tomography; MRI, magnetic resonance imaging; SPECT, single photon emission computed tomography; FDG-PET, Flurodeoxyglucose- positron emission tomography; EEG, electroencephalogram.

**Table 2:** Research criteria for the clinical diagnosis of probable or possible mild cognitive impairment due to Lewy body dementia (MCI-LB).

**Essential for a diagnosis of MCI-LB** is MCI defined by the presence of each of the following: 1. Concern by the patient, informant, or clinician regarding cognitive decline. 2. Objective evidence of impairment in one or more cognitive domains. The cognitive impairment may include any domain, but is more likely to be associated with attention-executive and/or visual processing deficits. 3. Preserved or minimally affected performance of previously attained independence in functional abilities, which do not meet the criteria for dementia.
**Core clinical features:** 1. Fluctuating cognition with variations in attention and alertness. 2. Recurrent visual hallucinations. 3. REM Behavior disorder. 4. One or more spontaneous cardinal features of parkinsonism: bradykinesia, rest tremor, or rigidity.
**Supportive clinical features:** Severe sensitivity to antipsychotic agents; postural instability; repeated falls; syncope or other transient episodes of unresponsiveness; prolonged or recurrent delirium; autonomic dysfunction, e.g., constipation, orthostatic hypotension, urinary incontinence; hypersomnia; hyposmia; hallucinations in other modalities including passage, and sense of presence phenomena; systematized delusions; apathy, anxiety, and depression.
**Proposed biomarkers** a. Reduced dopamine transporter uptake in basal ganglia demonstrated by SPECT or PET. b. Polysomnographic confirmation of REM sleep without atonia. c. Reduced meta-iodobenzylguanidine (MIBG) uptake on myocardial scintigraphy
**Potential biomarkers of MCI-LB:** a. Quantitative EEG showing slowing and dominant frequency variability. b. Relative preservation of medial temporal lobe structures on structural imaging. c. Insular thinning and gray matter volume loss on MRI. d. Low occipital uptake on perfusion/metabolism scan.
**Note:** MCI plus supportive clinical features or potential biomarkers are insufficient to diagnose MCI-LB but may raise suspicion of it and prompt biomarker investigation, which may add weight to an existing MCI-LB diagnosis. MCI-LB is less likely in the presence of any other physical illness or brain disease including cerebrovascular disease, and sufficient to account in part or in total for the clinical picture, although these do not exclude an MCI-LB diagnosis and may serve to indicate mixed or multiple pathologies contributing to the clinical presentation.
Probable or possible mild cognitive impairment due to Lewy body dementia (MCI-LB): • Probable MCI-LB can be diagnosed if: Two or more core clinical features of DLB are present, or only one core clinical feature is present, but with one or more proposed biomarkers. Probable MCI should not be diagnosed based on biomarkers alone. • Possible MCI-LB can be diagnosed if: Only one core clinical feature of DLB is present, with no proposed biomarkers, or one or more of the proposed biomarkers is present, but there are no core clinical features.

Adapted from Mckeith et al. [18].

**Table 3:** Summary of the overlap and dissimilarities in the biomarker findings of PDD, DLB, and AD.

Biomarkers	PDD versus DLB	PDD/DLB versus AD
**MRI**	**VBM:** Diffuse cortical atrophy in both PDD and DLB; gray matter reductions in the temporal, parietal and occipital lobes in DLB [93,102]. **DTI:** controversial, corticostriatal disruption in PDD is more frontal and in DLB more posterior (parietal and occipital) [39,102,120]. **SWI:** Nigrosome 1 “swallow tail sign” differentiates PD from controls [124]. No studies comparing DLB from PDD. **QSM:** Higher iron load in hippocampus, thalamus, parietal, frontal, and occipital cortices correlate with cognition in PD [127,128,129,130,131,132,133]. No studies in DLB. **fMRI:** No conclusive differences between PDD and DLB [95,139,140].	**VBM:** Insular cortical thinning may differentiate DBL from AD [99,100]. Preserved hippocampal volumes in DBL differentiate from AD [14]. **DTI:** Posterior regions affected in DLB more than AD [116]. **SWI:** Nigrosome 1 “swallow tail sign” differentiates DLB from AD with sensitivity (80%) [125]. **QSM:** Positive associations between susceptibility and amyloid PET in the pallidum and putamen of AD [134]. No studies in DLB. **fMRI:** No major differences between DLB and AD groups [138].
**PET and SPECT**	**DAT:** Reduced dopamine transporter uptake in PD and DLB. **SPECT perfusion and FDG-PET:** Similar perfusion profiles in PDD and DLB, with posterior hypoperfusion and hypometabolism (inferior parietal and occipital cortices) [149,152]. More prominent hypometabolism in the anterior cingulate in DLB than PDD [148].	**DAT:** Reduced striatal DAT uptake in DLB, useful in the differential diagnosis from AD, sensitivity (78%), and specificity (90%) [145]. **SPECT perfusion and FDG-PET:** Greater hypoperfusion in the parietooccipital cortex in DLB and PDD compared to AD [147]. Occipital hypometabolism combined with a less prominent metabolic decline in the medial temporal lobe in DBL vs. AD [148]. The “cingulate island” sign has high sensitivity (100%) in differentiating DLB from AD [14].
**PET amyloid and tau**	**amyloid:** Increasing amyloid pathology PD < PD-MCI < PDD < DLB. Positive amyloid PET is associated with worse global cognition in LBD [161,162,163,172]. **tau:** Under investigation, no conclusive data.	**amyloid:** Positive amyloid PET indicative of amyloid pathology (AD or co-pathology with LBD) **tau:** Under investigation, no conclusive data.
**[^123^I]MIBG Scintigraphy**	Lower uptake in DLB than those with PDD [154], but the latest can also be positive.	Would help to differentiate AD from LBD [21,156,157].
**Molecular fluid biomarkers**	**CSF:** (1) Low CSF Aβ_42_ levels predict cognitive impairment in PD and DLB [98,109,186,187,188,189]. Oxidized α-helical form of Aβ_1-40_ peptide significantly increased in patients with DLB in comparison to PDD [191]. (2) Lower α-syn in DLB and PD than in controls [193,196,208]. It may not be useful to differentiate between PDD and DLB [208]. (3) Other biomarkers: Inflammatory factors may increase in PDD and DLB but may not differentiate them [218]. NfL was also elevated in DLB, but no significant differences compared to PDD [213,216].	**CSF:** (1) AD pattern (low Aβ_42_, high t-tau and p-tau): AD > DLB > PDD > PD [191]. (2) α- oligomeric α-syn higher in DLB and PDD compared with AD and controls [195,197,205], also at prodromal stages [207] (3) Other biomarkers: unspecific elevation of NfL levels in DLB, which do not distinguish from other dementias [213].
**Other potential biomarkers**	**Microglia activation:** Increased 11C-PK11195 binding in several regions in PDD and DLB [178,179,180,181], but still under investigation (no conclusive data).	**Microglia activation:** Under investigation, no conclusive data.

PDD, Parkinson disease dementia; DLB, Dementia with Lewy bodies; VBM, Voxel-based morphometry; DTI, Diffusion Tensor Imaging; SWI, susceptibility-weighted imaging; QSM, quantitative susceptibility mapping; fMRI, functional magnetic resonance imaging; DAT, dopamine transporter; SPECT, single-photon emission computed tomography; PET-FDG, F-18 fluoro-deoxy-glucose positron emission tomography; (^123^I-MIBG), ^123^I-metaiodobenzylguanidine; CSF, cerebrospinal fluid.

## Data Availability

Not applicable.
